# Simulation and nurse-mentoring in a statewide nurse mentoring program in Bihar, India: diagnosis of postpartum hemorrhage and intrapartum asphyxia

**DOI:** 10.12688/gatesopenres.13490.1

**Published:** 2022-06-13

**Authors:** Rakesh Ghosh, Susanna Cohen, Hilary Spindler, Divya Vincent, Mona Sterling, Aritra Das, Aboli Gore, Tanmay Mahapatra, Dilys Walker

**Affiliations:** 1 Global Health Sciences, University of California, San Francisco, San Francisco, CA, San Francisco, 94158, USA; 2 College of Nursing, University of Utah, 10 South 2000 East, Salt Lake City, UT, 84112, USA; 3 Obstetrics and Neonatal, PRONTO India, State RMNCH, AG Colony, Patna, 800025, India; 4 Concurrent Monitoring learning and Evaluation, CARE India, 14 Patliputra Colony, Patna, Bihar, 800013, India; 5 Capacity Building, CARE India, 14 Patliputra Colony, Patna, Bihar, 800013, India; 6 Department of Obstetrics and Gynecology and Reproductive Services, University of California, San Francisco, San Francisco, CA, 94110, USA

**Keywords:** Mentorship, birth asphyxia, non-vigorous infant, maternal health, neonatal health, low- and middle-income countries

## Abstract

**Background**: Mentoring programs that include simulation, bedside mentoring, and didactic components are becoming increasingly popular to improve quality. These programs are designed with little evidence to inform the optimal composition of mentoring activities that would yield the greatest impact on provider skills and patient outcomes. We examined the association of number of maternal and neonatal emergency simulations performed with the diagnosis of postpartum hemorrhage (PPH) and intrapartum asphyxia in real patients.

**Methods:** We used a prospective cohort and births were compared between- and within-facility over time. Setting included 320 public facilities in the state of Bihar, India May 2015 – 2017. The participants were deliveries and livebirths. The interventions carried out were mobile nurse-mentoring program with simulations, teamwork and communication activities, didactic teaching, demonstrations of clinical procedures and bedside mentoring including conducting deliveries. Nurse mentor pairs visited each facility for one week, covering four facilities over a four-week period, for seven to nine consecutive months. The outcome measures were diagnosis of PPH and intrapartum asphyxia.

**Results:**Relative to the bottom one-third facilities that performed the fewest maternal simulations, facilities in the middle one-third group diagnosed 26% (incidence rate ratio [IRR] = 1.26, 95% confidence interval [CI]: 1.00, 1.59) more cases of PPH in real patients. Similarly, facilities in the middle one-third group, diagnosed 25% (IRR = 1.25, 95% CI: 1.04, 1.50) more cases of intrapartum asphyxia relative to the bottom third group that did the fewest neonatal simulations. Facilities in the top one-third group (i.e., performed the most simulations) did not have a significant difference in diagnosis of both outcomes, relative to the bottom one-third group.

**Results:**Relative to the bottom one-third facilities that performed the fewest maternal simulations, facilities in the middle one-third group diagnosed 26% (incidence rate ratio [IRR] = 1.26, 95% confidence interval [CI]: 1.00, 1.59) more cases of PPH in real patients. Similarly, facilities in the middle one-third group, diagnosed 25% (IRR = 1.25, 95% CI: 1.04, 1.50) more cases of intrapartum asphyxia relative to the bottom third group that did the fewest neonatal simulations. Facilities in the top one-third group (i.e., performed the most simulations) did not have a significant difference in diagnosis of both outcomes, relative to the bottom one-third group.

**Conclusions:** Findings suggest a complex relationship between performing simulations and opportunities for direct practice with patients, and there may be an optimal balance in performing the two that would maximize diagnosis of PPH and intrapartum asphyxia.

## Introduction

Capacity building of frontline health workers continues to be a priority for improving the quality of maternal and newborn care, particularly in low-resource settings ^1– 4^. Many of these programs combine simulations with bedside mentoring, didactic sessions, and demonstrations of clinical procedures. Studies from both high- and low-resource settings not only show that simulation-based training of healthcare providers can contribute to the acquisition and retention of clinical skills, but also improve patient outcomes ^5– 10^. Simulation training in low-resource settings takes a variety of forms (from one-off trainings to low-dose, high-frequency courses as well as center-based to near-situ and *in-situ* approaches). Established programs like Helping Mothers Survive (HMS) Bleeding After Birth have reported a link between skills and drills and decreased incidence of postpartum hemorrhage (PPH) induced complications ^9, 11^. Likewise, the Helping Babies Breathe (HBB) simulation program has shown increased adherence to neonatal resuscitation algorithms including stimulation and suctioning of the depressed neonate ^3^. Evidence show that simulation-based training contributes to improvement in clinical behaviors (including the performance of bag-mask ventilation), but a decay over time has also been observed ^12^. Thus, there is a need to understand *what type of* and *how much* simulation training leads to optimum use of evidence based practices (EBPs) thereby improving patient care and outcomes ^13^. A large scale nurse-mentoring program with integrated simulation, implemented in the eastern Indian state of Bihar, presented a unique opportunity to investigate this research question.

With a population of over 100 million, of which about 80% is rural, Bihar is one of the impoverished places in South Asia ^14, 15^. Despite improvements, the state is amongst the worst performing in India with maternal and child health indicators languishing below national averages ^16^. In 2016, the maternal mortality ratio in the state was 165 per 100,000 livebirths and the infant mortality rate was 38 per 1000 livebirths ^16^. Two leading causes of maternal and infant mortality are PPH and intrapartum asphyxia, respectively ^17, 18^.

To improve the quality of obstetric and newborn care, in 2011, CARE-India, a non-governmental organization, collaborated with the state Government of Bihar and implemented a range of initiatives through a pilot program in eight districts of Bihar. Inspired by the success of the pilot initiative ^19^, it was expanded to all 38 districts of the state as a nurse-mentoring program called AMANAT or ‘Apatkaleen Matritva evam Navjat Tatparta’ (meaning readiness for emergency obstetrical and neonatal care). The overarching goal of the AMANAT program was to reduce maternal and neonatal mortality, by improving quality of delivery and newborn care through didactic lessons, bedside mentoring, simulation, and teamwork and communication (T&C) activities. This was done through *in-situ*, on-the-job mentoring of labor and delivery (L&D) care providers using low tech, high-fidelity materials.

In two previous publications, we have reported: (1) increase in uptake of EBPs in normal non-complicated deliveries, and (2) overall improvement in diagnosis and management of PPH and intrapartum asphyxia in complicated deliveries, in the AMANAT program ^20, 21^. In this investigation, we examined the association of the number of maternal and neonatal emergency simulations conducted in the training program with diagnosis of PPH and intrapartum asphyxia in real patients. This analysis focuses on simulation, which is one specific component of the complete AMANAT intervention program. We used the SQUIRE 2.0 guidelines from the EQUATOR Network to report this quality improvement study ^22^.

## Methods

### Ethics statement

The study was approved by the Institutional Committee for Ethics and Review of Health Management Research Office of the Indian Institute of Health Management Research in Jaipur, India and the Committee for Human Research at the University of California San Francisco. Study ID# 14-15446.

### Study intervention and personnel

The AMANAT program was implemented in four phases in 320 Basic Emergency Obstetric and Neonatal Care (BEmONC) facilities between May 2015 and January 2017. Each phase covered 80 facilities, which were chosen based on facility readiness to implement the program. Since the inception of the AMANAT nurse-mentoring program, PRONTO International has worked with CARE-India to integrate simulation and team-training into the AMANAT curriculum, tailored to address local contextual needs.

During the AMANAT program, 120 mentors (with Bachelor's degree) were trained in simulation facilitation and debriefing best-practices ^23^. They received two trainings, simulation facilitation and advanced simulation facilitation training that were roughly four months apart. After the first training, a pair of mentors visited one facility per week covering four assigned facilities in about a month. The nurse-mentor pairs repeated their visits every month to their assigned facilities. In other words, a facility received one week of mentoring per month for seven to nine consecutive months. The AMANAT mentors trained the L&D team in the facilities where each team comprises of one to two nurses with or without any other birth attendant. Physicians or specialists are rarely available. The nurses are either Auxiliary Nurse Midwives with a 2-year training on multipurpose community health or General Nurses with a 3-year training in general nursing and midwifery. The details of the AMANAT program and PRONTO’s simulation and team-training have been elaborated elsewhere and are briefly described below ^20, 21, 24^.

Embedded in the AMANAT program was PRONTO’s curriculum that included simulations to promote the use of EBPs, improve T&C, and to increase awareness around person-centered maternity care among L&D staff. To improve T&C between providers, PRONTO’s curriculum includes TeamSTEPPS™ ^25^ concepts within its simulation exercises resulting in a curriculum, which allowed nurses to practice both technical and non-technical skills in a simulated scenario that replicates a real emergency. Mentor pairs facilitated simulations and debriefs, both of which were video recorded ^26^. In addition to simulation and T&C activities, mentors were encouraged to guide and supervise clinical care of patients in real time from admission to discharge, hereafter referred to as bedside mentoring. Finally, there were regular knowledge reviews (case-based learning) and routine didactic sessions with demonstrations on topics such as the partograph, active management of third stage of labor, infection control, fetal heart rate monitoring, basic resuscitation, golden minute for an asphyxiated newborn, and kangaroo mother care. More contextual information is presented in the *Extended data* ^27, 28^.

### Data sources

For this analysis we used data from two separate sources that are designed, collected and managed by CARE’s Concurrent Measurement and Learning team: (1) the Facility Information System (FIS), a web-based routine monitoring system used to record data on live deliveries and training activities during the weeks of mentoring; and (2) the direct observation of deliveries (DOD) before and after implementation of the AMANAT intervention. A third source, maintained by UCSF/PRONTO, recorded the number of simulations performed, which was used to validate the FIS based simulation counts. Data on select delivery practices were collected on all deliveries, daily, by mentors using observations and registers during their visits to the facilities and subsequently entered into the FIS. For the DOD, clinically trained project personnel visited each facility for a week to observe real deliveries occurring between 9 AM and 5 PM, approximately. The DOD provided comprehensive information on specific EBPs for individual deliveries both before and after the intervention periods. Data for this analysis was obtained from the FIS because it provided sufficient sample size to power the investigation on rare complications. The DOD data was used to generate facility level practice scores to adjust the final models, as described elsewhere ^21^.

### Clinical outcomes

The two clinical complications investigated in this analysis were diagnosis of maternal PPH and intrapartum (or birth) asphyxia of the newborn as defined by the World Health Organization ^29, 30^. PPH was defined as blood loss associated with obstetric labor or childbirth of more than 500ml for a vaginal delivery. Intrapartum (or birth) asphyxia was defined as failure by the neonate to initiate or sustain breathing at birth.

### Statistical analysis

We used two metrices to quantify the association between performance of simulations and diagnosis of complications. The first metric was total counts of maternal or neonatal complication simulations that were conducted in the entire mentoring period in a facility. The facilities were ranked based on the number of simulations performed and grouped into three roughly equal categories (tertiles): bottom-third, middle-third and top-third. The second metric was the ratios of the count of simulations to the count of other activities (T&C, demonstrations of various clinical skills, bedside mentoring and didactic lessons), performed in the facilities. Therefore, we had four ratios – (1) simulations : T&C, (2) simulations : demonstrations, (3) simulations : bedside mentoring and (4) simulations : didactic lessons. Like the first metric, each of these ratios were categorized into three ordered groups (tertiles). For example, a facility in the top-third sim: didactic ratio group spent more time doing simulations relative to didactic sessions than a facility in the bottom-third group (reference group). The four ratios were estimated separately for maternal complication simulations and neonatal complication simulations. We examined both continuous form and categories of simulations. A small percentage (~1%) of the facilities had high values of these ratios, meaning the number of simulations performed were much higher compared to the other activities. To minimize the outlier effect and to avoid imposing linearity in the relationship, we generated tertiles from the continuous ratios, using 33 ^rd^ and 66 ^th^ percentiles as cut-offs. Programmatically, results in categories are more meaningful to implementors as it will provide a specific number (midpoint of the range) or range of simulations associated with maximum improvement. In contrast, analysis using a linear continuous form (of simulation counts) will not provide a maximum limit, which aside from being infeasible will imply more simulations is better. This might not always be the case as it may take away time from other important activities, including conducting mentored real deliveries.

The analytical strategy used for this analysis is similar to the one previously reported ^20^. Births and complications between the start and end date of each mentoring week for each facility were aggregated. Thus, one row of observation represented one week in one facility. We used negative binomial models appropriate for the count of complications. To account for excessive zeros, i.e., no complications in a facility in a week, we utilized the zero-inflated negative binomial model and reported the incidence rate ratios (IRRs) with 95% confidence intervals (CIs). The IRRs compared the middle-third group of facilities with the bottom-third group and the top-third with the bottom-third, for both metrices. For readers interested in statistical details, we have rationalized the choice of models in the supplement and in a previous publication ^20^. Deliveries were clustered in both time and space, so we used the sandwich variance estimator, which provides appropriate standard errors after accounting for non-independence of observations ^31^.

The final models were adjusted for weeks of nurse-mentoring, days per week of nurse-mentoring, total number of births per week, number of T&C activities performed, phase of the AMANAT program, availability of physicians, facility level practice scores and proportion of mentee-sessions attended. Mentee-sessions attended were defined as follows: if facility A had 10 mentees and week 1 of mentoring had 40 total sessions, there will be a maximum of 400 mentee-sessions in that facility(A)-week(1). Of these, if 8 mentees were present for all 40 sessions, 9 ^th^ mentee was present for 30 sessions, and 10 ^th^ mentee was present for 10 sessions only, this would equate to 90% mentee-sessions attendance [(320+30+10)/400] for that facility(A)-week(1).

Facility level practice scores were generated from the DOD data using a set of 11 intrapartum and 12 newborn care indicators, explained in a prior publication ^21^. Individual indicators were assigned a score of 1 if they were performed as recommended; otherwise, a 0 was assigned. Individual delivery scores were rescaled to range between 0 and 100, where 0 refers to none and 100 refers to all EBPs being performed appropriately for the delivery. The indicator-specific scores were aggregated to obtain a delivery score and scores for all deliveries in a facility were averaged to obtain a facility level practice score.

Significance was examined at the 5% (two-tailed) level. As the categories of simulations were ordered, we performed a chi-squared linear test of trend to examine increasing or decreasing trend in the associations ^32^. Analysis was done using STATA 16.1. Patients or public *were not* involved in the design, conduct, or reporting or dissemination plans of the research.

## Results

In total, there were 55,938 live deliveries that occurred in the 320 facilities during the weeks of mentoring, of which PPH was identified in 1291 (2%) and intrapartum asphyxia in 1631 (3%) cases ^27^. During the mentoring period, the median (IQR: interquartile range) number of maternal and neonatal complication simulations performed per facility were 18 (12 – 24) and 9 (6 – 12), respectively. In the same period, the median number of T&C activities performed per facility was 5 (3 – 11). Median (IQR) ratios of maternal complication simulations equaled: Sim: T&C activities, 0.17 (0.10 – 0.26); Sim: demonstrations, 0.46 (0.25 – 0.89); Sim: bedside mentoring, 0.10 (0.04 – 0.21); and Sim: didactic lessons, 0.21 (0.12 – 0.38). The median (IQR) ratios of neonatal complication simulations equaled: Sim: T&C activities, 0.08 (0.05 – 0.13); Sim: demonstrations, 0.24 (0.13 – 0.46); Sim: bedside mentoring, 0.05 (0.02 – 0.10); and Sim: didactic sessions, 0.11 (0.06 – 0.19).

### Postpartum hemorrhage

**Figure 1.**
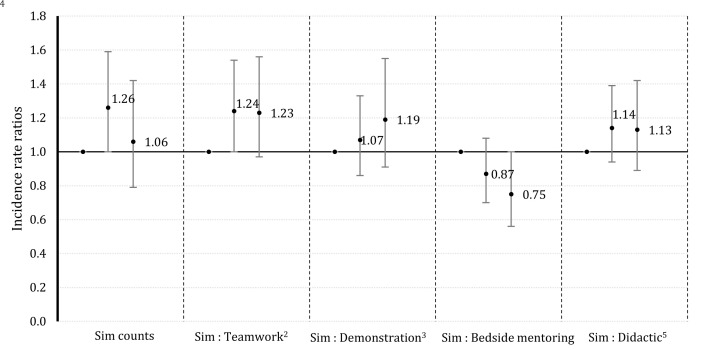
Adjusted1 incidence rate ratios for the diagnosis of postpartum hemorrhage with simulation counts or ratios of simulations performed relative to other activities in the AMANAT nurse-mentoring program in Bihar, India (2015 – 2017). ^1^ Adjusted for weeks of nurse-mentoring, days per week of nurse-mentoring, total number of births per week, phase of program, availability of physicians, proportion of mentee-sessions attended, facility level practice. ^2 3 5^ These associations represent the added benefit of doing a simulation relative to doing a teamwork activity. Likewise, for a demonstration activity or a didactic session. ^4^ The decreased association for the ratio, simulation: bedside mentoring, shows that a proper mix of the two is more beneficial than just performing simulation. *Note: The solid dots represent the associations, and the vertical bars represent the upper and the lower confidence intervals.*

Relative to the bottom-third facilities that did fewest (~9) maternal complication simulations, facilities in the middle one-third group (~19) diagnosed 26% (IRR = 1.26, 95% CI: 1.00, 1.59) more cases of PPH in real deliveries, while the top one-third (~31 sims) did not show increased diagnosis ( Figure 1). This association between maternal complication simulation and PPH diagnosis increased to 1.33 (95% CI: 1.04, 1.70) after co-adjustment for T&C activities, demonstration sessions, bedside mentoring and didactic sessions. Analysis using the ratio metric of the number of maternal complication simulations to the number of T&C activities shows facilities in tertile 2 diagnosed 24% (IRR = 1.24, 95% CI: 1.00, 1.54) more PPH cases, compared to the facilities in tertile 1 (those that performed fewest maternal complication simulations relative to T&C activities) ( Figure 1). Facilities in tertile 2, spent about twice as much time performing simulations relative to T&C activities, then the facilities in tertile 1. In contrast, performing more simulations relative to bedside mentoring, decreased diagnosis of PPH because facilities in tertile 3 diagnosed 25% (IRR = 0.75, 95% CI: 0.56, 1.00) fewer PPH cases, compared to facilities in tertile 1 ( Figure 1). Facilities in tertile 3 did about 5 maternal complication simulations for every 10 live deliveries, whereas facilities in tertile 1 did about 2 simulations for every 10 live deliveries. The elevated but non-significant point estimates generally suggest that performing maternal complication simulations relative to didactic lessons may increase PPH diagnosis.

### Intrapartum asphyxia

**Figure 2.**
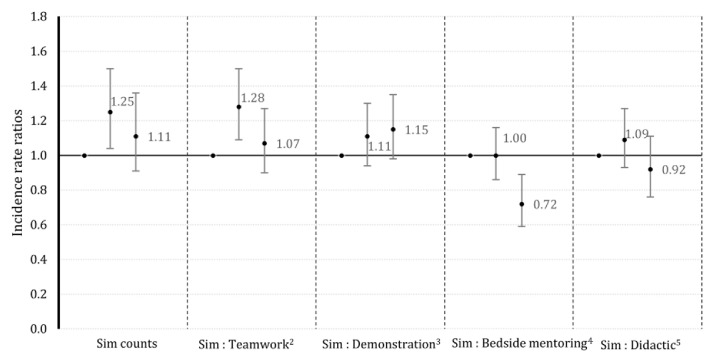
Adjusted1 incidence rate ratios for the diagnosis of intrapartum asphyxia with simulation counts or ratios of simulations performed relative to other activities in the AMANAT nurse-mentoring program in Bihar, India (2015 – 2017). ^1^ Adjusted for weeks of nurse-mentoring, days per week of nurse-mentoring, total number of births per week, phase of program, availability of physicians, proportion of mentee-sessions attended, facility level practice. ^2 3 5^ These associations represent the added benefit of doing a simulation relative to doing a teamwork activity. Likewise, for a demonstration activity or a didactic session. ^4^ The decreased association for the ratio, simulation: bedside mentoring, shows that a proper mix of the two is more beneficial than just performing simulation. *Note: The solid dots represent the associations, and the vertical bars represent the upper and the lower confidence intervals.*

For intrapartum asphyxia, facilities in the middle one-third group (did about 9 neonatal complication simulations), diagnosed 25% (IRR = 1.25, 95% CI: 1.04, 1.50) more cases relative to the bottom-third facilities (~4) that conducted fewest neonatal simulations ( Figure 2), which changed to 1.22 (95% CI: 1.01, 1.47) after co-adjustment with counts of T&C activities, demonstration sessions, bedside mentoring and didactic sessions. Facilities that did the highest (~ 16) number of neonatal simulations showed no improvement in diagnosis, with no linear trend across groups ( Figure 2). Results also suggest that performing neonatal complication simulations relative to T&C activities increase diagnosis of intrapartum asphyxia ( Figure 2). Facilities in tertile 2, with a moderate number of neonatal complication simulations performed relative to T&C activities, diagnosed 28% (IRR = 1.28, 95% CI: 1.09, 1.50) more cases of intrapartum asphyxia, compared to the facilities in tertile 1 ( Figure 2). Facilities in tertile 2, spent more than twice as much time performing simulations than T&C activities, then the facilities in tertile 1. The elevated but non-significant point estimates suggest performing neonatal complication simulations relative to demonstrations activities may also increase diagnosis of intrapartum asphyxia. In contrast, performing more neonatal complication simulations relative to bedside mentoring, decreased diagnosis of intrapartum asphyxia. For example, facilities in tertile 3 diagnosed 28% (IRR = 0.72, 95% CI: 0.59, 0.89) less intrapartum asphyxia cases, compared to the facilities in tertile 1, with a significant linear decreasing trend ( Figure 2). Facilities in tertile 3 did about 1 neonatal complication simulation for every 5 live deliveries, whereas facilities in tertile 1 did about 1 simulation for every 50 live deliveries.

There was no linear relationship between any of the metrices and both outcomes. The full models are presented in supplementary Tables 1 and 2 and the numerical estimates for Figure 1 and Figure 2 are presented in supplementary Tables 3 and 4 (see *Extended data* ^27, 28^).

## Discussion

The results suggest a positive association between performance of maternal complication simulations and diagnosis of PPH as well as between performance of neonatal complication simulations and diagnosis of intrapartum asphyxia. However, diagnosis increased with performance of simulations up to a certain point, beyond which it did not appear to increase diagnosis, as evidenced by no linear association and no association for tertile 1. In fact, time spent in simulation relative to other activities is likely important in diagnosing these complications. For example, we found that performing maternal and neonatal complication simulations is relatively more beneficial than performing T&C activities. However, bedside mentoring also appears to be important and an optimal balance between performing simulated deliveries and bedside mentoring is likely to be most effective in teaching diagnosis of complications. Thus, there appears to be a complex relationship between performing simulations and opportunities for hands-on practice with real patients. Striking a right balance that is appropriate for the setting is key to providing quality care.

Facilities that performed the highest number of simulations did not achieve statistically significant improvement in diagnosis compared to those that did the fewest. This could be because the fidelity of simulations or the following debriefs may have been compromised due to the volume. However, previous reports do not support this explanation, in general; whether the same is true in the high simulation facilities, cannot be unequivocally said ^33, 34^. Alternatively, performing too many simulations could be a diversion from other activities including bedside mentoring, which as results suggest is important to clinical learning. 

There is no study to our knowledge to which these results can be directly compared because of the unique setting in which simulation is integrated (not stand-alone) into a comprehensive nurse mentoring intervention. A systematic review reported insufficient evidence to suggest that simulation training improves neonatal resuscitation ^3^. However, other reports from HBB simulation training reported improved clinical performance of stimulation, suction, and bag-mask ventilation; and demonstrated positive impact on fresh stillbirth and mortality on the first day of life ^1, 2, 10, 35^. Evidence on retention of knowledge and skills after simulation training is mixed ^2, 36^. A cluster randomized trial conducted in Kenya and Uganda demonstrated the PRONTO intervention was effective in reducing intrapartum stillbirth and early neonatal mortality in preterm gestations ^37^. In Mexico, a modular stand-alone PRONTO training was able to reduce cesarean sections and neonatal mortality ^38^ and in Guatemala, the intervention increased the use of EBPs ^39^. A previous AMANAT program based study analyzed direct observation of live deliveries and demonstrated an association between performance of simulations and increased use of EBPs in non-complicated births ^21^.

These findings are of interest to the global simulation community that continues to design and implement the right mix of capacity strengthening strategies to improve quality of delivery and newborn care. The study brings scientific evidence from a large-scale initiative with variability in the dose of program components and their potential impact. Consequently, this lends limited external validity to similar facilities in low-resource settings that possess comparable levels of readiness, infrastructure and staff. The longitudinal nature of the investigation, the large sample size and the use of causal inference methods give some confidence that these results are less likely to be biased. Associations with simulations were relatively robust to adjustments for T&C activities, demonstration sessions, bedside mentoring and didactic sessions, when all were included in the same model. Nevertheless, the possibility of residual confounding from unmeasured factors cannot be ruled out. Evidence of validity of PPH and intrapartum asphyxia diagnosis in this study is empirically demonstrated by the associations observed with established risk factors. For example, PPH was associated with obstructed or prolonged labor (1.36, 95% CI: 1.08, 1.73) and anemia (1.43, 95% CI: 1.22, 1.68), while intrapartum asphyxia was associated with multiple births (1.20, 95% CI: 1.06, 1.36), cord prolapse (1.38, 95% CI: 1.02, 1.87), breech presentation (1.35, 95% CI: 1.19, 1.54) and anemia (1.19, 95% CI: 1.09, 1.30).

## Limitations

There are several limitations, important among which was the absence of a control group. We cannot state if similar changes concurrently happened in facilities where the AMANAT intervention was not implemented. We could not investigate other complications such as hypertensive disorders of pregnancy, infections etc. since we did not have sufficient data. The global incidence of PPH is thought to be around 6% and that of intrapartum asphyxia is around 5–10% ^40, 41^. Thus, the two outcomes in this study were under-reported. According to the principle of regression dilution bias, if under-reporting of outcomes are non-differential in relation to the number of simulations performed, the statistical significance will be affected and not the point estimate ^42^. The under-reporting could be differential but there is no way to make that assessment. The actual change in diagnosis could have been due to a host of contextual factors not accounted for in the models. For example, when the monsoons arrive after summer, some districts (equivalent of a US county) in the state get flooded. Mentors in the affected places accordingly adjusted the curriculum. Facility preparedness and change in district or state level leadership, which depended on political dispensation, also affected the implementation of the program. Teasing out specific change that is due to simulation or other factors is difficult given the integrated and vast nature of the AMANAT program. Finally, several statistical tests were performed at the 5% significance level, giving rise to the possibility of some results to be statistically significant by chance. We did not adjust for multiple comparison because we were pursuing a well laid out *a-priori* hypothesis and not randomly searching for significant associations.

## Conclusions

The study provides rare evidence on the effectiveness and dosage of simulation from a rural and impoverished area in South Asia. We found that a moderate number of *in-situ* simulations tailored to address the local needs, balanced with real-time bedside mentoring may be the formula suited to improving the diagnosis of PPH and intrapartum asphyxia in livebirths.

## Data availability

### Underlying data

Dryad: Data for ‘Simulation and nurse-mentoring in a statewide nurse mentoring program in Bihar, India: diagnosis of postpartum hemorrhage and intrapartum asphyxia’. https://doi.org/10.7272/Q6VQ30X9 ^27^.

This project contains the following underlying data:

- FIS_births_complication_counts_long_wk1&7.dta

### Extended data

Zenodo: Simulation and nurse-mentoring in a statewide nurse mentoring program in Bihar, India: diagnosis of postpartum hemorrhage and intrapartum asphyxia. https://doi.org/10.5281/zenodo.6584800 ^28^.

This project contains the following extended data:

- Online_supplement_01062021.docx (Supplementary methods and Tables 1–4)

Data are available under the terms of the Creative Commons Zero "No rights reserved" data waiver (CC0 1.0 Public domain dedication).
