# Supplementary material for: Simulation and nurse-mentoring in a statewide nurse mentoring program in Bihar, India: diagnosis of postpartum hemorrhage and intrapartum asphyxia
Source: Gates Open Res. 2022 Jun 13;6:70. [Version 1] doi: 10.12688/gatesopenres.13490.1 (PMC10616110; doi:10.12688/gatesopenres.13490.1)
Supplement: Peer review history

# Reviewer report: Reviewer response for version 1

**DOI:** 10.21956/gatesopenres.14752.r35041

**Published:** 2023-10-30

**Authors:** Antoinette du Preez

**Affiliations:** 1 North-West University, Potchefstroom, South Africa

This is a very good article and it is well written. My only comments or questions are as follows:

1. Why is the APGAR score not included?
2. I wonder was the age profile link on a demographic basis?
3. How many of these asphixia babies received breastfeeding?

Well done.

Is the work clearly and accurately presented and does it cite the current literature?

Yes

If applicable, is the statistical analysis and its interpretation appropriate?

I cannot comment. A qualified statistician is required.

Are all the source data underlying the results available to ensure full reproducibility?

Yes

Is the study design appropriate and is the work technically sound?

Yes

Are the conclusions drawn adequately supported by the results?

Yes

Are sufficient details of methods and analysis provided to allow replication by others?

Yes

Reviewer Expertise:

Midwifery lecturer at the North-West University (Potchefstroom) South Africa an Advanced midwife.

I confirm that I have read this submission and believe that I have an appropriate level of expertise to confirm that it is of an acceptable scientific standard.

# Reviewer report: Reviewer response for version 1

**DOI:** 10.21956/gatesopenres.14752.r35252

**Published:** 2023-10-23

**Authors:** Babar Hasan

**Affiliations:** 1 Sindh Institute of Urology and Transplantation, Karachi, Pakistan

This article tried to look at the real-world effectiveness of one component of a nursing mentorship program (AMANAT: a combination of simulation, team work and communication activities, didactic teaching and bed side mentoring) in effecting the ability of a nurse in diagnosing post-partum hemorrhage and birth asphyxia. The authors demonstrated that the correlation between quantity of exposure to emergency situation simulation and desired outcome is complex and may not be truly linear. A combination of the right number of simulation along with bedside mentoring may lead to better training.

 General comments:

 Overall this is a very well written and thought out study. The methodology and analysis is sound. I have a couple of observations which if answered will be helpful to further add to the robustness of the study:

1. How was the individual competency of the mentors assessed? Mentoring (especially bedside mentoring) is an art and variability in individual mentorship skills can have an influence on how individual mentees learn and perform (even if it is simulation-based teaching).
2. Was there continuous professional development of mentors? If yes, how often?
3. Was DOD used to assess mentors competency too? If yes, can the authors provide some details on that?
4. How was the quality of simulation teaching assessed for each mentor? If yes, how was it controlled for as a confounder?
5. Was any attempt made in doing inter-observer variability/agreement between FIR and DOD assessments?
6. Seems like overall the median simulation to other activities ratio is quite low (overall simulations sessions were low), do the authors think that they have missed the ‘true’ simulation number threshold due to such low number of simulation sessions overall?

Is the work clearly and accurately presented and does it cite the current literature?

Yes

If applicable, is the statistical analysis and its interpretation appropriate?

Yes

Are all the source data underlying the results available to ensure full reproducibility?

Partly

Is the study design appropriate and is the work technically sound?

Yes

Are the conclusions drawn adequately supported by the results?

Yes

Are sufficient details of methods and analysis provided to allow replication by others?

Partly

Reviewer Expertise:

Congenital heart disease, quality improvement, human centred mentorship, employee engagement, Artificial intelligence

I confirm that I have read this submission and believe that I have an appropriate level of expertise to confirm that it is of an acceptable scientific standard.

# Reviewer report: Reviewer response for version 1

**DOI:** 10.21956/gatesopenres.14752.r32703

**Published:** 2022-11-08

**Authors:** Krishnamurthy Jayanna

**Affiliations:** 1 M S Ramaiah University of Applied Sciences, Bangalore, Karnataka, India

The authors have made a good attempt at using data from multiple sources to arrive at how the interventions have impacted clinical care. Role of simulations and how they complement traditional training systems in improving quality of care is relevant today in LMIC that are striving hard to fast track MNCH-related SDGs.

 However, the results do not show any particular pattern. This could be due to two reasons: 1) there is no linear relationship between the simulations and quality, more so in primary health care centers in LMIC where there are many other factors that may influence the results. 2) Secondly, is it to do with the methodology and statistical analyses used in the study? A methodological and a statistical expert may be better positioned to comment on this.

 The results aren't conclusive and hence do not add significantly to existing knowledge base related to interventions to improve QoC. However the conclusions in the main paper seem more definitive and conclusive, when compared to how they appear in the abstract. The latter seems more aligned with the evidence from this study. The conclusions in the main paper should be revisited to reflect the actual findings from the study.

Is the work clearly and accurately presented and does it cite the current literature?

Yes

If applicable, is the statistical analysis and its interpretation appropriate?

I cannot comment. A qualified statistician is required.

Are all the source data underlying the results available to ensure full reproducibility?

Yes

Is the study design appropriate and is the work technically sound?

Partly

Are the conclusions drawn adequately supported by the results?

Partly

Are sufficient details of methods and analysis provided to allow replication by others?

Yes

Reviewer Expertise:

Maternal and Neonatal Health, Quality of Care, Health Systems Strengthening, Implementation Science and Implementation Research

I confirm that I have read this submission and believe that I have an appropriate level of expertise to confirm that it is of an acceptable scientific standard, however I have significant reservations, as outlined above.
